# Feasibility of atrial fibrillation ablation via right internal jugular access using a lattice-tip pulsed field ablation system in the presence of inferior vena cava aplasia: first clinical experience

**DOI:** 10.1093/ehjcr/ytag541

**Published:** 2026-07-17

**Authors:** Dimitrios Gerontitis, Dimitrios Katsaras, Apostolos Katsivas, Panagiotis Ioannidis

**Affiliations:** Heart Rhythm Center, IASO Hospital, 37-39 Kifissias Avenue, Athens 151 23, Greece; Heart Rhythm Center, IASO Hospital, 37-39 Kifissias Avenue, Athens 151 23, Greece; Heart Rhythm Center, IASO Hospital, 37-39 Kifissias Avenue, Athens 151 23, Greece; Heart Rhythm Center, IASO Hospital, 37-39 Kifissias Avenue, Athens 151 23, Greece

## Case description

**Figure 1:**
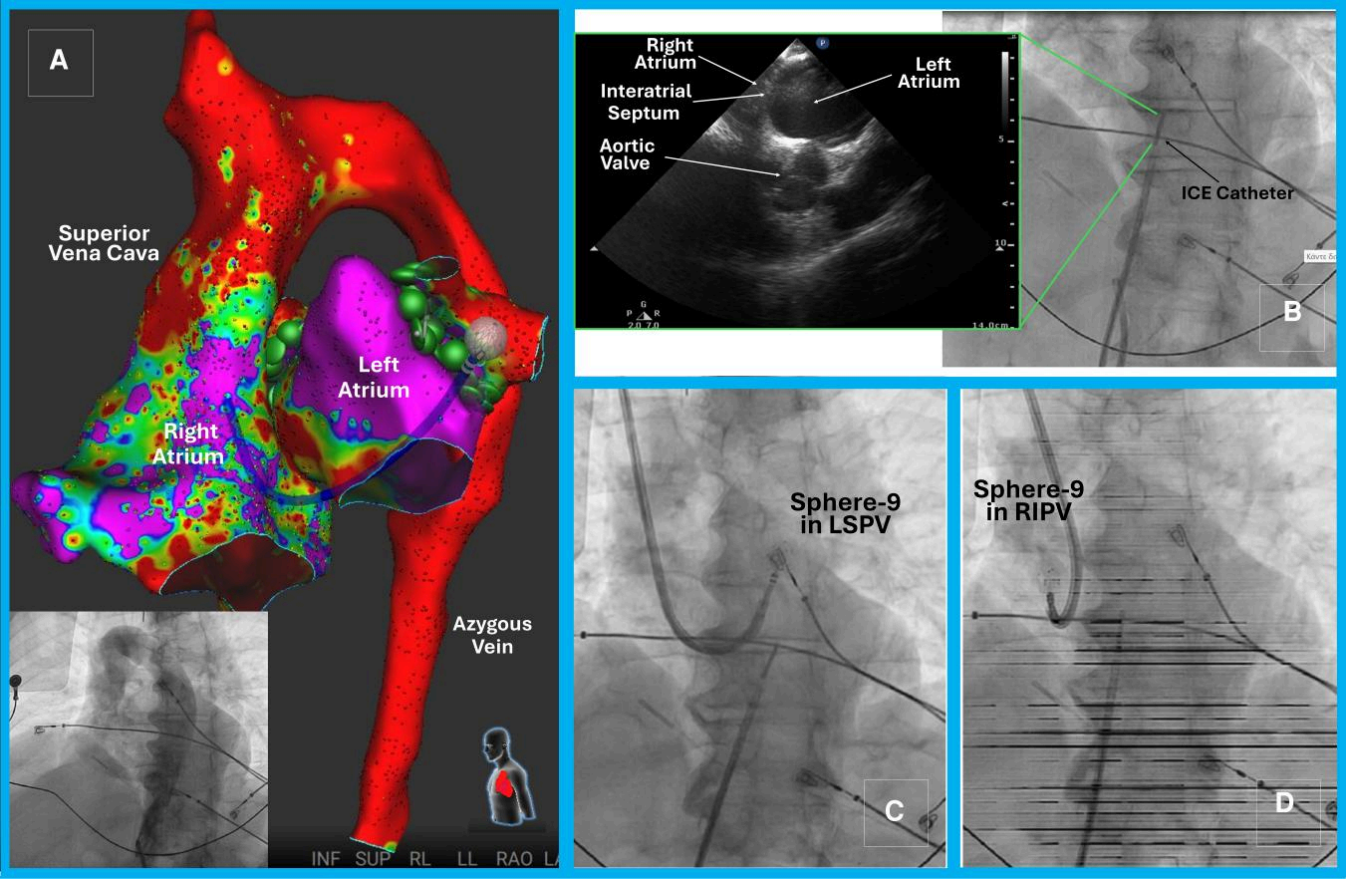
Procedural imaging and navigational approach. (*A*) Three-dimensional electroanatomical reconstruction (Affera Prism-1™) demonstrating complete inferior vena cava aplasia with venous drainage diverted via a dilated azygous vein into the superior vena cava; the inset provides a fluoroscopic angiogram confirming the anomalous venous trajectory. (*B*) Intracardiac echocardiography visualization from the azygous vein (posterior vantage point) showing the interatrial septum, fossa ovalis, and adjacent aortic valve, facilitating safe transseptal puncture from the superior approach. (*C*, *D*) Fluoroscopic views (left anterior oblique projections) showing the Sphere-9™ lattice-tip catheter positioned at the antrum of the left superior pulmonary vein and right inferior pulmonary vein via the Agilis™ steerable sheath, introduced through the right internal jugular vein.

A 75-year-old male with symptomatic paroxysmal atrial fibrillation and underlying sinus bradycardia was scheduled for pulmonary vein isolation. Initial femoral venous access revealed complete inferior vena cava (IVC) aplasia, with venous return diverted via a dilated azygous vein into the superior vena cava (*Figure 1A*, Supplementary material online, *Videos S1*–S3).

To circumvent the anatomical obstacle, the procedure was converted to a right internal jugular vein (RIJV) approach. Crucially, a ViewFlex™ Xtra (Abbott, St. Paul, MN, USA) intracardiac echocardiography (ICE) probe was advanced from the femoral vein into the dilated azygous vein, providing an optimal posterior view for visualizing the interatrial septum and fossa ovalis (*Figure 1B*, Supplementary material online, *Video S4*). Following ultrasound-guided RIJV puncture, a Preface™ transseptal sheath (Biosense Webster, Irvine, CA, USA) was introduced. Under continuous azygous-ICE guidance, transseptal puncture was performed using a BRK™ needle (Abbott) with a manually exaggerated curve.^1^ The Preface™ sheath was then exchanged over a wire for an Agilis™ (Abbott) steerable sheath to access the left atrium. High-density mapping and ablation were performed using the Affera Prism-1™ system and the Sphere-9™ (Medtronic, Minneapolis, MN, USA) lattice-tip catheter, which was introduced through the Agilis™ sheath.

The catheter’s bidirectional deflection, in combination with the Agilis™ sheath guidance, allowed for stable tissue contact despite the challenging superior approach (*Figure 1C*, *1D*). Circumferential antral ablation was performed using pulsed-field energy. Successful isolation of all pulmonary veins was confirmed by post-ablation mapping, demonstrating complete electrical isolation. The patient was discharged the following day in stable sinus rhythm and remained arrhythmia-free during short-term follow-up. Post-procedural CT imaging (see Supplementary material online, *Video S5*) confirmed infrarenal IVC aplasia with azygous continuation, independent hepatic vein drainage directly into the right atrium, intestinal malrotation, and multiple splenules, diagnostic of heterotaxy (polysplenia) syndrome.^2^

While transjugular access has been historically utilized to overcome femoral venous obstacles, to our knowledge, this represents the first report of AF ablation via RIJV access using the specific lattice-tip PFA platform. This case highlights the system's safety and effectiveness when conventional femoral access is precluded by complex venous anatomy.

## Supplementary Material

ytag541_Supplementary_Data

## Data Availability

The data underlying this article are available in the article and in its online Supplementary material. Further inquiries can be directed to the corresponding author.

## Notes

**Consent:** The authors confirm that written informed consent was obtained from the patient for the publication of this case report and accompanying images in accordance with COPE guidelines. The signed consent form is held by the treating institution.
